# The Impact of EFL Learners’ Negative Emotional Orientations on (Un)Willingness to Communicate in In-person and Online L2 Learning Contexts

**DOI:** 10.1007/s10936-024-10071-y

**Published:** 2024-03-07

**Authors:** Mehdi Solhi

**Affiliations:** Department of English Language Teaching, School of Education, Istanbul Medipol University, Istanbul, Turkey

**Keywords:** L2 boredom, L2 anxiety, Demotivation, L2WTC

## Abstract

The present study explored how negative emotional orientations (i.e., anxiety, boredom, and demotivation) may contribute to English as a foreign language (EFL) learners’ willingness to communicate (WTC) in in-person and online classes. In doing so, a total of 290 university students majoring in English were recruited to fill in a set of scales. The structural equation modeling analysis indicated that foreign language classroom anxiety (FLA) and L2 demotivation have a direct impact on EFL learners’ in-person and online L2WTC. While L2 demotivation was the strongest significant predictor of learners’ in-person L2WTC, FLA was the strongest predictor of online L2WTC. Additionally, there was a positive correlation among FLA, L2 demotivation, and foreign language classroom boredom (FLB). While FLA demonstrated no direct impact on communication willingness, it exhibited significant indirect paths to in-person L2WTC via the full mediation of L2 demotivation and FLA . Although the result did not show any significant direct impact of FLB on online L2WTC, it had a small yet significant indirect path to online L2WTC through the full mediation of FLA. FLA also revealed indirect significant paths to online L2WTC through FLB and L2 demotivation. The implications for L2 teachers and teacher educators will be further discussed.

## Introduction

Practicing oral communication is crucial for learning a second or foreign language (L2), especially when opportunities for communication outside the classroom are limited (Harmer, 2015; Peng, 2019). However, incorporating communicative speaking practices in the classroom setting might be challenging due to variations in L2 students’ willingness to participate, which is likely to be influenced by various psychological, linguistic, and sociological factors as well personality traits (Khajavy et al., 2018; Lan et al., 2021; Peng, 2015; Piechurska-Kuciel, 2018; Syed & Kuzborska, 2020; Wei & Xu, 2022). Consequently, L2 learners’ reluctance to engage in communication opportunities and practices in the class, which is generally known as unwillingness to communicate, can arise from a complex interplay of negative emotional dispositions and learner characteristics. This aligns with MacIntyre et al.’s (1998) model of willingness to communicate (WTC), in which communication willingness represents the final stage before actual L2 use, highlighting L2WTC is highly susceptible to affective orientations and motivational propensities.

A few recent studies have identified several negative affective orientations, including foreign language classroom boredom (FLB), foreign language classroom anxiety (FLA), and L2 demotivation, as factors that can significantly impact L2WTC (Khajavy et al., 2018; Peng, 2015; Zhang et al., 2020, 2022). Although the negative impact of FLB on L2 learning has been acknowledged (Pawlak et al., 2022), there has been limited attention given to the potential influence of FLB on L2WTC (Zhang et al., 2022). Given FLB can affect L2 students’ cognitive processing and attention (Derakhshan, Kruk, et al., 2022; Kruk et al., 2021; Pawlak et al., 2021), it is likely to make it more difficult for them to focus on and process language input, as well as generate appropriate responses in communicative practices in L2 class (Pan et al., 2023). This, in turn, can hinder their willingness to actively engage in oral communication and meaningful interactions with their peers and the teacher in in-person classes. The construct of FLB has also been indicated to be prevalent in online class and a handful of research highlights the antecedents of this negative affective orientation in virtual classes (e.g., Derakhshan et al., 2021; Kruk et al., 2022). Since L2 learners who experience higher levels of boredom in general are likely to be affected even more in online L2 learning contexts (Chen et al., 2022), investigating the way FLB is likely to influence their WTC in both online and in-person classes appears to be an intriguing research avenue.

FLA is another affective disposition that has been indicated to be closely associated with FLB (Dewaele et al., 2023; Li & Han, 2022; Li & Wei, 2023). This negative emotional disposition is generally considered to influence L2 performance (Dörneyi, 2005) and decreases L2WTC (Khajavy et al., 2018). Indeed, FLA is among the most influential factors negatively associated with L2WTC (Elahi Shirvan et al., 2019; Peng, 2015). The impact of FLA on online L2WTC has also been aknowledged in L2 studies (e.g., Alqarni, 2021; Lee et al., 2021). More specifically, L2 speaking anxiety significantly predicts EFL learners’ L2WTC in both in-person and online classes (Mulyono & Saskia, 2021). The presence of FLA is likely to create a barrier to effective communication by triggering feelings of apprehension and worry, hindering L2 learners’ confidence and thereby their willingness to actively participate in speaking activities in both online and face to face classes.

Another important construct, closely associated with FLB and FLA is L2 demotivation (El Deen, 2023; Gao & Liu, 2022). Identified as arguably a greater issue in L2 learning than motivation (Thorner & Kikuchi, 2019), this multifaceted construct has recently attracted the attention of L2 researchers worldwide (Chong et al., 2019). Studies have identified several internal and external factors which contribute to L2 learners’ demotivation (e.g., Dörnyei, 1998; Kikuchi, 2015; Unal & Yanpar Yelken, 2016). Drawing upon Pekrun et al.’s (2010) control-value theory of achievement, both too much and too little control by L2 teacher can be demotivating factors which can negatively impact students’ sense of control, self-efficacy, and feelings (Oxford, 2001). The inadequacy of educational facilities and material, negative attitudes, and reduced self-confidence are among the other factors that are likely to trigger L2 demotivation (Dörnyei, 1998). Kikuchi (2015) also identified several categories of internal and external L2 demotivators that can negatively influence L2 learning. They include teacher behaviors, characteristics of classroom, classroom environment, learning materials, experience of failure, and loss of interest. Despite a wealth of research on demotivating factors in L2 learning (e.g., Dörnyei, 1998; Kikuchi, 2015; Oxford, 2001; Zhang et al., 2020), studies on L2 demotivation remains underexplored (Dörnyei & Ushioda, 2021) and more specifically there is a scarcity of research on the way L2 demotivation can specifically influence L2WTC (Eddy-U, 2015). It is plausible to argue that L2 demotivation can adversely affect L2 learners’ willingness to communicate by diminishing their intrinsic motivation, reducing their engagement in language activities, and undermining their confidence in using the L2 in in-person classes.

While several studies have examined the connection between L2WTC and motivation in online education (e.g., Balouchi et al., 2021; Balouchi & Samad, 2021; Freiermuth & Jarrell, 2006; Mulyono & Saskia, 2021), there is a noticeable gap in research regarding the relationship between L2WTC and demotivation in the context of online L2 education. In general, limited research has explored the construct of L2 demotivation in online education (e.g., Iftanti et al., 2023; Wu et al., 2020). Exploring how L2 demotivation can adversely influence communication willingness of EFL learners in online education thus appears to be a fruitful research path.

In the present study, it is thus hypothesized that negative affective dispositions including FLB, FLA, L2 demotivation can negatively influence L2WTC in in-person and online classes. Despite the acknowledged interplays between these affective orientations, there remains a paucity of research that explores them altogether in one single study, and how they are correlated with L2WTC in different educational contexts. This study contributes to L2 literature by exploring the potential relationships between FLB, FLA, L2 demotivation, and WTC, a facet that has not been thoroughly taken into scrutiny in previous L2 studies.

## Literature Review

### L2 Boredom

L2 boredom, or FLB as conceptualized by Li et al. (2021a), is a multifaceted emotional disposition that has recently been introduced as an intriguing topic of inquiry in L2 studies (Kruk, 2016; Pawlak et al., 2020a, 2023). FLB as an L2 domain-specific construct is defined “as a negative emotion with an extremely low degree of activation/arousal that arises from ongoing activities […] (that) are typically over-challenging or under-challenging” (Li et al.,2021a, p. 12). In other words, this emotional orientation is characterized by the absence of stimulation or arousal in L2 practices that are either excessively or insufficiently challenging. Additionally, stimulation or arousal that are void of meaning, relevance, or significance to L2 learners are likely to trigger FLB. Studies have indicated several internal and external antecedents of FLB. For example, L2 teacher behaviors characterized by excessive teacher control or inadequate support, lack of linguistic proficiency, task characteristics, and materials have been found to account for FLB in the class (Solhi, Derakhshan et al., 2023; Nakamura et al., 2021; Pawlak et al., 2023).

In addition to the factors that contribute to FLB, this emotional state has been affirmed to adversely influence various aspects of language learning and classroom dynamics, including L2 academic achievement (Dewaele et al., 2023; Zhao & Wang, 2023), L2 resilience (Alrabai & Alamer, 2022), motivated behavior (Pawlak et al., 2022), L2 engagement (Derakhshan, Fathi et al., 2022), and L2WTC (Zhang et al., 2022). For example, in Dewaele et al.’s (2023) study with a cohort of EFL learners, FLB was predictive of L2 academic achievement, with FLA indicating the stronger negative correlation with L2 achievement. Given there is a significant and positive correlation between L2 achievement and L2WTC (Al-Murtadha, 2021), it can be inferred that FLB may affect L2WTC through their impact on L2 academic achievement. Indeed, a close association between FLB and L2WTC has been found in Zhang et al.’s (2022) study with undergraduate EFL university students. Their study indicated that FLB significantly and directly influences EFL students’ WTC in the class.

The experience of FLB in online education has also been taken into scrutiny in a strand of L2 research (e.g., Chen et al., 2022; Derakhshan et al., 2021; Kruk et al., 2022; Solhi & Önen, 2023). In Chen et al.’s (2022) study with L2 university learners, inappropriate teaching techniques (e.g., insufficient explanation and fast speaking pace), learning contents (e.g., a heavy load of homework), and violating learners’ perception (e.g., implementing traditional teaching methods) were the antecedents of FLB in the context of online L2 learning. In Chen et al.’s (2022) study, insufficient opportunity to communicate in online classes was among the antecedents of FLB, highlighting the negative impact of limited interaction on individuals’ WTC in L2 learning process. In Derakhshan et al.’s (2021) study with a group of EFL university students, lack of student participation was identified as one of the main antecedents of FLB. The finding implies that lack of student engagement and active involvement in L2 communications can contribute to a decrease in their WTC, as the limited opportunities for participation and interaction may hinder the development of their communicative skills and confidence in using the target language.

Despite the infamous impact of FLB on various aspects of L2 learning (Solhi et al., 2023), a few studies have found contradicting results in terms of the positive associations between FLB and consistency of interest (Pawlak et al., 2022) and L2WTC (Wang et al., 2021). In Pawlak and his colleagues’ (2022) study, English majoring university students’ FLB positively predicted consistency of interest. The researchers ascribe this finding to the dimensional model of boredom, highlighting that this emotional state can have both deactivating and activating functions. Put simply, higher levels of boredom may lead to increased arousal and a desire for change which can motivate participants to seek novel learning directions. Consequently, rather than losing interest in L2 learning, they strategically focus on other aspects of L2 learning to maintain their interest. Wang et al.’s (2021) study with the undergraduate students majoring in English indicated a positive relationship between boredom and L2WTC. According to the researchers, one possible explanation is that when classroom activities lack opportunities for student engagement, L2 learners who desire to communicate with their teachers and peers may be more inclined to participate to create a more interactive learning environment. The authors also ascribe the finding to personality traits such as extroversion that is likely to contribute to both boredom and WTC. In a nutshell, considering the dimensional model of boredom, it appears to be interesting to examine how the experiences of FLB is different online and in-person classroom context can impact EFL learners’ L2WTC.

### L2 Anxiety

FLA is an L2 domain-specific negative affective orientation which pertains to L2 learners’ inclinations to feel anxious when using or learning an L2 (Dewaele et al., 2023). The construct was originally conceptualized by Horwitz et al. (1986, p. 128) as a “distinct complex of self-perceptions, beliefs, feelings, and behaviors related to classroom language learning arising from the uniqueness of the language learning process”. FLA is so prevalent in L2 context and a significant number of students at each proficiency level experience anxiety when engaging in English classroom speaking activities (Liu, 2006). That is why despite a wealth of studies on the construct of FLA, it continues to be a significant concern for L2 teachers and an appealing topic of inquiry for L2 researchers (Dewaele et al., 2023).

A strand of research has identified the self-related antecedents of FLA, indicating that it is susceptible to, say, self-perceived L2 competence (Cheng, 2002), self-perceived L2 achievement (Luo, 2018), self-perceived low control of L2 learning (Yang et al., 2021). Indeed, the studies highlight that FLA is highly prone to individual factors, and L2 learners’ self-perceptions have a major impact on their FLA experiences. In Yang et al.’s (2021) study, L2 learners’ lower ability of self-regulation was also identified as another individual contributor that leads to negative emotions including FLA. MacIntyre and Charos (1996) similarly suggest that individuals who have lower levels of emotional stability may be more susceptible to experiencing L2 anxiety. The association between FLA and personality traits such as neuroticism has also been identified in L2 research (e.g., Dewaele, 2013). In sum, a wealth of studies indicates that different from positive emotional orientations such as L2 enjoyment which is strongly associated with external factors such as teacher, FLA is strongly linked to the student-internal factors (e.g., Dewaele et al., 2018; Yuan et al., 2023). However, along with the internal factors that influence FLA in the class, learner-external factors such as classroom environment can have an influential impact on FLA experiences (e.g., Li et al., 2021a).

The associations between FLA and social context have also been taken into scrutiny in L2 studies. For example, In Jiang and Dewaele’s (2020) research with more than one thousand university students, the participants reported that speaking English with friends elicited the least anxiety, whereas speaking English in public was perceived as the most anxiety-inducing situation. This aligns with Liu’s (2006) findings, which indicated that having oral communication in English as a pair work is less FLA-provoking that responding to the teacher or speaking English in front of the class. In fact, these findings suggest that the social context can significantly impact FLA during English speaking activities.

FLA has also been indicated to be negatively predictive of L2 achievement (Tahmouresi & Papi, 2021) and L2 proficiency (Alrabai, 2022). The negative impact of FLA on L2WTC has also been confirmed by a strand of L2 studies (Dewaele, 2019; Fathi et al., 2021; Peng, 2015; Peng & Woodrow, 2010). For instance, Dewaele’s (2019) study indicated that FLA was the strongest predictor of L2WTC among EFL learners. In their study, L2 enjoyment and teacher’s frequency of using the target language were identified as positive predictors of L2WTC. This is echoed in Peng’s (2015) research, which indicated that FLA was the strongest direct predictor of L2WTC inside the class, while L2WTC outside the class was not influenced by this emotional orientation. The insignificant impact of FLA on L2WTC outside the class suggests that additional contributing factors such as FLB and L2 demotivation might have a major role in influence L2WTC inside the class. Given these two affective orientations have been acknowledged to be closely associated with FLA (Dewaele et al., 2023; El Deen, 2023; Gao & Liu, 2022; Li & Wei, 2023), investigating the interplay between these emotional dispositions can contribute to a deeper understanding of the factors that influence L2WTC inside and outside the classroom so as to develop strategies for promoting effective communication in L2 learning process.

Furthermore, additional research is needed to explore and understand the distinct contributors of L2WTC in different contexts, including online education. Aligned with Pekrun’s (2006) control-value theory, in online education where L2 learners may possess a lower level of control over their own learning, they are more likely to experience higher levels of FLA than in-person classes. This, in turn, could negatively impact L2WTC in online classes. Thus, foreign language online classroom anxiety appears to be a context-bound and domain specific type of anxiety that is likely to impact L2WTC. Lee et al. (2021) found a negative correlation between FLA and L2WTC in autonomous English activities in out-of-class digital contexts. In their study, FLA mediated the relationship between L2WTC and informal digital learning of English, that is, self-directed English activities in informal digital settings driven by personal interests and conducted independently without teacher assessment (Lee & Lee, 2021). Interestingly, despite the autonomy offered by such online learning environments, individuals may still feel anxious while showing their willingness to communicate in L2. This highlights the ubiquitous nature of FLA in L2 learners’ communication experiences, even in informal digital settings. In the context of formal virtual environments, Alqarni (2021) also identified that L2 speaking anxiety had a negative impact on L2WTC in both in-person and online learning environments, with in-person classes showing a slightly stronger association.

### L2 Demotivation

Demotivation is characterized as the decline or loss of interest that was previously present (Brown, 2014). Dörnyei and Ushioda (2021) describe a demotivated L2 learner as someone who was once motivated but has since lost their interest or commitment in the process of L2 learning. L2 demotivation is a multifaceted construct that needs to be differentiated from amotivation. The latter refers to a complete absence of motivation, often stemming from feelings of incompetence and helplessness (Brown, 2014). L2 learners’ loss of motivation pertains to “various negative influences that cancel out existing motivation” (Dörnyei & Ushioda, 2021). They can be attributed to external factors such as insufficient school facilities, teacher personality and teaching methods, and uninteresting learning materials as well as internal factors such as negative attitudes and reduced self-confidence (Dörnyei, 1998). Kikuchi (2015) similarly identified various internal and external factors that contribute to L2 demotivation, including demotivating behaviors exhibited by teachers, characteristics of the classroom, the materials used in the classroom, experiences of failure, and loss of interest in the subject matter (see Instruments). Gao and Liu (2022) also classify L2 demotivators as learner-related factors (e.g., reduced self-confidence and FLA) as well as external forces (e.g., teacher, curriculum, and environment). In Albalawi and Al-Hoorie’s (2021) qualitative and longitudinal study, L2 university students’ fixed language learning mindset (LLM) was found as the primary factor that directly and indirectly contributes to L2 demotivation. Fixed LLM refers to an individual’s implicit theories and beliefs that L2 learning is an innate ability and cannot be changed through effort (Mercer & Ryan, 2010).

L2 demotivation has been acknowledged to be also closely associated with other psychological and educational factors, including teacher behavior (Pishghadam et al., 2021), L2 engagement (Zhang et al., 2020), L2 resilience (Kim et al., 2017), FLA (Choi, 2017; Gao & Liu, 2022), FLB (El Deen, 2023), and L2WTC (Eddy-U, 2015). For example, Zhang et al.’s (2020) study with EFL university students indicated FLA can play a mediating role between several dimensions of L2 demotivation (i.e., experience with failures, classroom materials, classroom characteristics, and demotivating teacher behavior) and their intention to continue the courses. The finding highlights the complex interplay between affective factors and (de)motivational processes in L2 learning. In their study, classroom engagement exhibited a mediating impact on the relationship between three subconstructs of L2 demotivation, namely loss of interest, classroom characteristics, and demotivating teacher behavior, and L2 achievement. In other words, when L2 learners become demotivated as result of the abovementioned factors, they are less likely to actively get engaged in L2 learning and this can consequently impact their achievement. In a different study on the association between L2 demotivation and FLA, Choi (2017) examined how Korean students perceive FLA and L2 motivation in the context of learning Japanese. The findings indicated a strong association between L2 demotivation due to peer pressure and FLA when speaking in Japanese. Additionally, FLA related to face-threatening situations was closely linked to L2 students’ motivation in learning Japanese. Indeed, this study highlights the way L2 demotivation and FLA can influence L2 learners’ WTC and oral communication in L2 learning.

The close association between L2 demotivation and FLB has recently captured the interest of L2 researchers, and FLB has been identified as factor which is associated with demotivation in L2 learning (Gao & Liu, 2022; Kikuchi, 2015). However, research on the link between these two constructs is scant (Pawlak et al.,2020a). In one of the few studies on this association by El Deen (2023), EFL university students’ L2 demotivation was identified as one of the antecedents of FLB. Indeed, there is a need for further research on the relationships between the constructs so as to better understand how they impact L2WTC in both online and in-person education. Additionally, as aforementioned, there is also a trade-off between FLA and L2 demotivation, and identifying the way these two negative orientations work in tandem to influence L2WTC appears to be an important research area that warrants further exploration. Apparently, a wealth of research abounds on the antecedents of L2 demotivation (e.g., Dörnyei, 1998; Kikuchi, 2015; Oxford, 2001; Albalawi & Al-Hoorie’s (2021). However, despite the widespread occurrence of language-learning failure (Dörnyei & Ushioda, 2021), L2 demotivation remains a rather underexplored area in L2 research, and more specifically there is a scarcity of research on the way L2 demotivation can influence L2WTC (Eddy-U, 2015).

Despite a body of research on L2 demotivation in in-person classes, there are only a few studies on the construct in online education. For example, Wu et al.’s (2020) study indicated that implementation of online flipped writing instruction is an effective approach to cope with L2 demotivation in English writing endeavors. Iftanti et al. (2023) identified several L2 demotivators in online education, encompassing aspects such as supporting facilities, peers, and learning environment. Apparently, there is a gap in literature on L2 demotivation and how it can impact willingness to communicate of EFL learners in online contexts.

### L2 Willingness to Communicate

L2WTC is generally defined as “the intention to initiate communication, given a choice” (MacIntyre et al., 2001, p. 369), and is characterized as “a dynamic situational variable” (Cao, 2014, p. 1). For the purpose of the current study, the construct is conceptualized as L2 learners’ dynamic predispositions to initiate or engage, mostly verbal, conversations in both in-person and online classroom contexts. Studies have identified various influential factors that can contribute to L2 learners’ WTC in in-person classes, including perception and attitudes toward the speaking activities (de Saint Léger & Storch, 2009), perseverance of effort and L2 enjoyment (Lee, 2022), L2 motivational attributes (Lee & Lee, 2020; Ma et al., 2019), and openness to experience (Piechurska-Kuciel, 2018). Along with the positive antecedents of L2WTC, studies have identified several deterrent forces that can thwart L2 learners’ communication willingness, encompassing disruptive environmental factors, lack of sense of relatedness, and insufficient communicative ability (Yarwood & Bennett, 2022). Lack of relatedness between peers was also identified as one of the antecedents of unwillingness to communicate in online classes (Fung, 2022; Yarwood & Bennett, 2022). The findings highlight the influential role of peer connectivity in forming L2 belongingness and consequently fostering L2WTC in online classes.

Apparently, the high level of communicative ability does not necessarily correspond with a high WTC disposition (Brown, 2014) and amalgamation of cognitive as well as affective orientations are in harness with L2WTC (Wang & Derakhshan, 2023). More specifically, FLB (Zhang et al., 2022), FLA (Khajavy et al., 2018; Peng, 2015), and L2 demotivation (Eddy-U, 2015) have been acknowledged to be associated with L2WTC. Zhang et al. (2022) investigated the association between EFL university students’ FLB and L2WTC. Results indicated that FLB significantly and negatively impacts L2WTC. In their study, FLB also had a mediating role between ideal L2 self and growth mindset, significantly influencing EFL learners’ WTC in L2 learning. Given that FLB is closely associated with (de)motivational attributes, identifying the complex interplay between these emotional states can provide valuable insights into understanding how they can influence L2WTC. In a study with a group of EFL university students, Eddy-U (2015) identified the demotivating forces that contribute to task-situated WTC. They include several internal aspects such as lack of confidence, lack of personal vision, inappropriateness for English level, and disinterest, as well as learner-external influences such as inappropriate classroom environment and bad groupmates. In Khajavy et al.’s (2018) study with more than one thousand five hundred secondary school students, FLA was negatively related to WTC, while L2 enjoyment exhibited a stronger association with L2WTC. The findings suggest that high levels of FLA can hinder students’ willingness and ability to engage in communication in L2 acquisition. As a result, students may be less likely to actively participate in L2 learning activities, which can ultimately impact their language proficiency and overall L2 learning experience. Khajavy and his colleagues’ (2018) findings are echoed by Peng’s (2015) study, which indicated that FLA is one of the strongest predictors of L2WTC inside the class. In Zhang et al.’s (2022) research, FLA similarly exhibited a mediating role between motivational attributes and L2WTC. Despite a few studies on L2WTC in the context of online education (e.g., Lee & Liu, 2022; Mulyono & Saskia, 2021), research on the complex interplay of this personality trait with negative affective orientations such as FLA, FLB, and L2 demotivation in virtual learning environments remains relatively underexplored. In one of the studies, Mulyono and Saskia (2021) investigated the impact of self-confidence, L2 speaking anxiety and motivation on EFL learners’ L2WTC in online environments. The participants were from secondary school and university students. The results indicated that all three affective variables significantly predicted EFL learners’ L2WTC in both in-person classes and online L2 learning settings.

In a nutshell, previous L2 research has shown limited focus to the impact of FLB on L2WTC (Zhang et al., 2022), and there is a scarcity of studies specifically examining how L2 demotivation affects L2WTC (Eddy-U, 2015). Additionally, considering the close association between FLA and L2WTC (Peng, 2015), in the current study, the researcher attempted to probe the associations between FLB, FLA, and L2 demotivation, and the extent they predict EFL learners’ L2WTC in online and in-person classes. In doing so, the following research questions were formulated:

#### Q1

What is the relationship between FLB, FLA, L2 demotivation, and L2WTC?

#### Q2

To what extent is L2WTC predicted by FLB, FLA, and L2 demotivation?

## Method

### Participants and Setting

The research sample for the present study consisted of 290 university students (Male = 102, 35.2%; Female = 188, 64.8%) majoring English language teaching (ELT) at various universities in Istanbul during the Fall 2022 semester (2022–2023 academic year). They were from different academic levels including freshmen (*N* = 98, 33.8%), sophomores (*N* = 97, 33.4%), juniors (*N* = 67, 23.1%), and seniors (*N* = 28, 9.7%). The mean age of the participants was approximately 21.70 years, ranging from 18 to 29. The undergraduate ELT programs in Turkey offer approximately 8–10 courses per academic semester, most of which are general English (e.g., oral communication, listening and pronunciation, reading and writing skills) in their first year, followed by more academic field-related courses (e.g., approached and methods in language education, linguistics, language and culture, critical reading and writing, and teaching language skills) over the following academic years. The program candidates are required to take the universities’ compulsory proficiency exam before being admitted to the department. Thus, they must attain a B1-B2 level in the standardized proficiency exams to be eligible for the enrollment. A questionnaire was administered to a voluntary cohort of EFL students to assess their affective orientations and L2WTC in both online and in-person classes.

### Instruments

#### L2 Boredom

L2 boredom was assessed by Li et al.'s (2021a) Foreign Language Learning Boredom questionnaire on a 5-point Likert scale. The scale consists of 7 components measuring state boredom and trait boredom in different learning contexts. In the present study, the items of the first factor measuring foreign language classroom boredom (8 items; e.g., *I start yawning in foreign language class because I am so bored*) were used to measure EFL learners’ boredom experiences in L2 classes.

#### L2 Anxiety

L2 anxiety was measured by Botes et al.’ (2022) developed and validated 8-item FLA questionnaire on a 5-point Likert scale. The scale aims to assess L2 domain-specific foreign language learning anxiety. Example items include *I can feel my heart pounding when I’m going to be called on in FL class* and *I start to panic when I have to speak without preparation in FL class*.

#### L2 Demotivation

L2 demotivation in the class was measured by modified Kikuchi’ (2015) L2 demotivation scale on a 4-point Likert scale ranging from 1 = Almost never true to 4 = Almost always true. The measure assesses internal and external demotivational factors that impact EFL learners’ learning experiences in the class. The six components of the scale include teacher behavior (4 items), characteristics of class (5 items), class environment (5 items), class materials (4 item), experience of failure (4 items), and loss of interest (4 items). Some minor revisions were made so as to more accurately measure EFL university learners’ L2 demotivation in the context of the class. For example, the word ‘readers’ was simply replaced with ‘materials’ in one of the items of the class materials component. Sample items include *I got low scores on tests such as midterm and final examinations* and *I lost my interest in English* from the subcomponents of experience of failure and loss of interest, respectively.

#### L2 WTC

L2WTC was assessed by Lee and Hsieh’s (2019) adopted in-class L2WTC scale which measures the extent the students are willing to communicate in English in in-person classes (4 items; e.g., *When you have a chance to talk in front of the other students in an English class*). In their study, values of Cronbach’s *α* (0.91) provided evidence of high internal consistency for the scale. The participants’ L2WTC in online classes were also measured using the same scale by simply adding “online” to “English class” so as to specifically measure L2WTC in online classes. I also made other minor changes such as replaying “in front of the other students” with “in the presence of other students” to better fit the context. The items were on a five-point Likert scale ranging from 1 = Definitely not willing to 5 = Definitely willing. Sample items on online class L2WTC include *When you have a chance to talk in the presence of other students in an online English class* and *When you have a group discussion in an online English class*.

### Data Collection Procedure

A set of questionnaires measuring their FLB, FLA, L2 demotivation, and L2WTC was administered to a cohort of EFL university students, and only the individuals who indicated a willingness to voluntarily participate in the study filled in the scales. The questionnaire was distributed both online and in-person to a group of instructors across various universities. They were requested to share it with students who expressed willingness to participate. Additionally, a snowball data collection technique was employed to reach more participants. They were instructed to consider their general affective orientations and WTC in English throughout the previous academic semester. The data collection was conducted in the last three weeks of the second academic semester, resulting in a total of 290 responses from the participants. Before commencing to respond the items, their consent was obtained through a consent form. They were also notified that their demographic information would be kept confidential.

### Data Analysis

Structural equation modeling (SEM) was used to investigate the associations between the constructs. In particular, the SEM model evaluated the role of FLA, FLA, and L2 motivation as predictors of L2 learners’ online and in-person L2WTC. In the present study, maximum likelihood (ML) was utilized as the estimation approach since it is resistant to unbiased estimate regardless of whether the constructs have slightly or moderately nonnormal distribution in large sample sizes (Hau & Marsch, 2004). Prior to the examination of the interdependence associations of a set of constructs in a structural model, the measurement model of these constructs was verified employing confirmatory factor analysis (CFA) (Hair et al., 1998). A number of model-fit indices was used to investigate the model fit. These included RMSEA 0.08, CFI > 0.90, TLI > 0.90, and SRMA 0.08 (see Hu & Bentler, 1995). The convergent validity of the variables was also assessed via the estimation of average variance extracted (AVE) (Hair et al., 1998). AVEs more than 0.50 showed the convergent validities of variables. To investigate the associations between components Pearson correlations were computed.

The indirect effects of FLA, FLA, and L2 motivation, as well as their combined effects on L2 learners’ online and in-person L2WTC, were examined using mediation analysis. Because the data had not been completely normalized, the path coefficients were examined using a 5,000-bootstrap analysis with a 95% confidence interval to see if the pathway values were within the interval’s range, thus normalizing the data (Kline, 2015). The bootstrap results showed that the model’s paths did not include zero and were within a 91% confidence interval. Finally, evidence for measurement invariance across genders (male vs. female) was determined using the following metrics: Δχ^2^*p* > .05, ΔCFI ≤ 0.010, ΔRMSEA ≤ 0.015, and ΔSRMR ≤ 0.03 (Cheung & Rensvold, 2002).

## Results

### Primarily Analyses

The CFA findings indicated that the measurement model fit the data well (2(1159) = 3273.98, *p* = .000, CFI = 0.915, TLI = 902, SRMR = 0.061, RMSEA = 0.052), indicating that it was acceptable for further investigation. All parameter estimations and standard errors were appropriate. All the loadings were significant (λ > 0.50). Standardized loadings for FLA ranged from 0.71 to 0.84, for FLB ranged from 0.71 to 0.87, for L2 demotivation ranged from 0.67 to 0.83 and for L2WTC ranged from 0.72 to 0.88. There were no notably large coefficients or standard errors. Most of absolute correlation residuals were less than 0.1, suggesting a satisfactory overall fit.

The reliability and convergent validity of the scales were also evaluated. The findings are demonstrated in Table 1. All four scales had appropriate level of α (> 0.70) and ꞷ(> 0.70). the AVEs were greater than 0.50, providing support for the convergent validity of constructs. To investigate the relationships between constructs, a correlation analysis was also done (see Table 2). Based on the findings, it was feasible to conclude that the psychometric properties of four scales were adequate for additional exploration.

**Table 1:** Descriptive statistics, reliability, convergent and discriminant validity of the constructs

	Mean	SD	Α	ꞷ	AVE
1. FLA	3.52	0.95	0.92	0.94	0.66
2. FLB	4.33	1.13	0.93	0.95	0.63
3. Demotivation	2.71	1.08	0.88	0.90	0.69
4. Teacher Behavior	2.23	1.83	0.84	0.87	0.65
5. Class Characteristics	2.85	1.74	0.72	0.75	0.52
6. Class Environment	2.64	1.68	0.71	0.73	0.56
7. Class Materials	2.66	1.82	0.81	0.82	0.58
8. Failure	3.01	1.51	0.82	0.84	0.53
9. Loss of Interest	2.72	1.88	0.77	0.79	0.59
10. In-person WTC	3.58	0.98	0.92	0.94	0.63
11. Online WTC	3.42	1.02	0.88	0.91	0.62

*Note* SD: Standard deviation α: Cronbach’s alpha; ω: Composite reliability; AVE: Average variance extracted; SD: Standard deviation

**Table 2:** Pearson’s correlation statistics for four scales

	1	2	3	4	5	6	7	8	9	10	11
1. FLA	-										
2. FLB	0.56^***^	-									
3. Demotivation	0.23^**^	0.51^***^	-								
4. Teacher Behavior	0.16^*^	0.55^***^	0.60^***^	-							
5. Class Characteristics	0.21^**^	0.52^***^	0.63^***^	0.32^***^	-						
6. Class Environment	0.20^**^	0.54^***^	0.72^***^	0.35^***^	0.48^***^	-					
7. Class Materials	0.16^*^	0.48^***^	0.73^***^	0.39^***^	0.47^***^	0.57^***^	-				
8. Experience of Failure	0.34^***^	0.55^***^	0.65^***^	0.18^**^	0.35^***^	0.35^***^	0.50^***^	-			
9. Loss of Interest	0.18^*^	0.59^***^	0.63^***^	0.25^**^	0.24^***^	0.33^***^	0.41^***^	0.52^***^	-		
10. In-person WTC	− 0.25^**^	− 0.12	− 0.41^***^	− 0.22^**^	− 0.27^***^	− 0.23^**^	− 0.26^**^	− 0.37^***^	− 0.39^***^	**-**	
11. Online WTC	− 0.37^***^	− 0.10	− 0.28^***^	− 0.19^**^	− 0.17^*^	− 0.20^**^	− 0.19^**^	− 0.30^***^	− 0.32^***^	0.61^***^	**-**

*Note* *:Significant at *p* < .05 **:Significant at *p* < .01, ***:Significant at *p* < .001

### The Direct Impacts of FLB, FLA, and L2 Demotivation on Online and in-person L2WTC

**Fig. 1:**
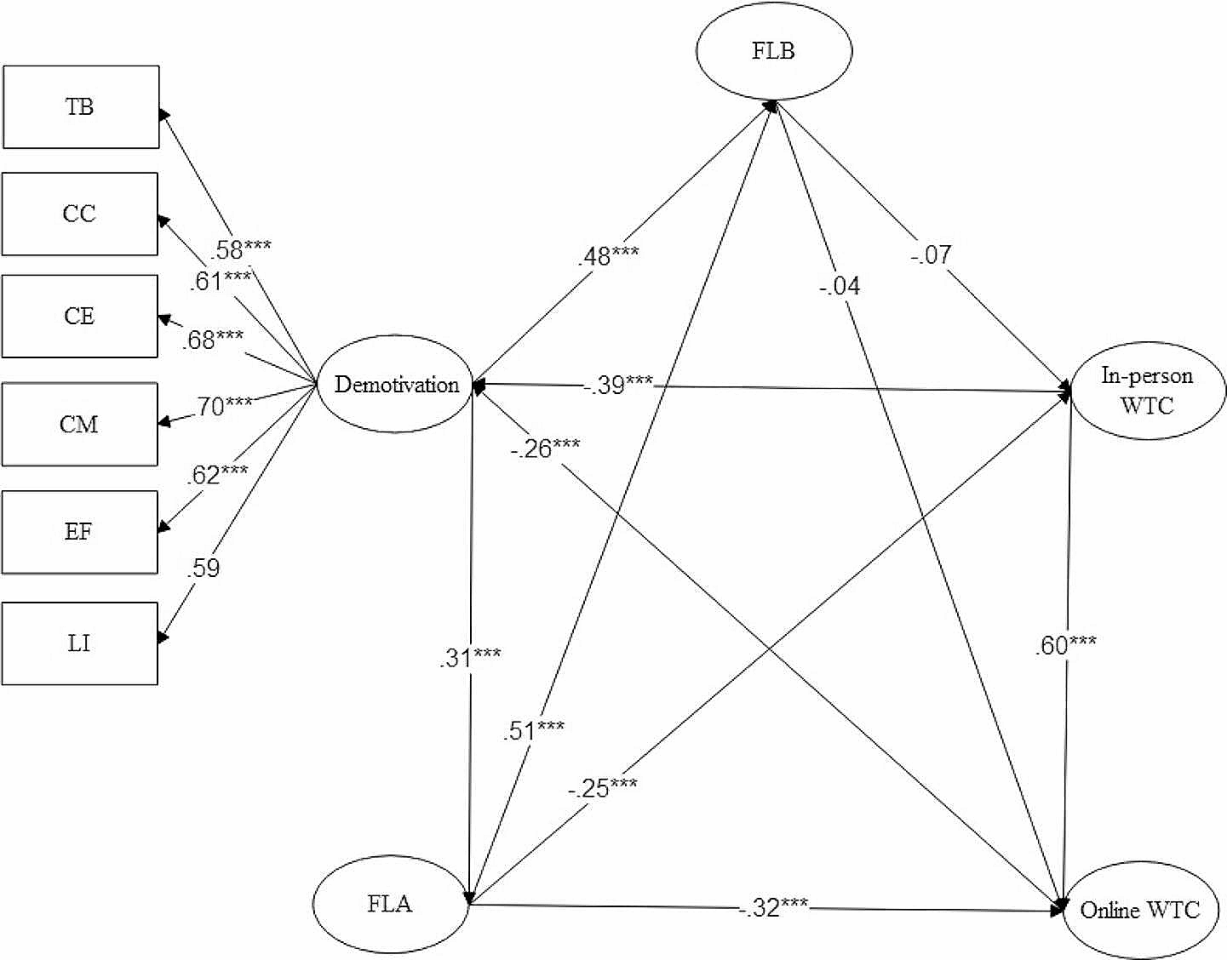
The structural model of the study. *L2WTC. Note*: FLA: Foreign Language Anxiety; FLB: Foreign Language Boredom; TB: Teacher Behavior; CC: Classroom Characteristics; CE: Classroom Environment; CM: Classroom Environment; EF: Experience of Failure; LI: Loss of Interest; WTC: Willingness to Communicate

Following the evaluation of the validity and model fit, the path coefficients were explored with regard to their significance and effect size. Figure 1and Table 3 show the direct impacts of the constructs in the model. According to the results, FLA and L2 demotivation have a direct impact on learners’ in-person and online L2WTC. However, there was no direct path from FLB to in-person and online L2WTC. With a rather moderate impact (β = − 0.391, p.001), L2 demotivation was the strongest significant predictor of learners’ in-person L2WTC. FLA also had a small impact on the in-person L2WTC (β = − 0.251, *p* < .001). With a relatively moderate size, FLA was the strongest predictor of learners’ online L2WTC (β = − 0.327, *p* < .001). L2 demotivation had a small effect on online L2WTC (β = -266, *p* < .001). The results also showed that FLB is significantly and positively linked to FLA (β = 0.391, *p* < .001) and L2 demotivation (β = 0.482, *p* < .001). FLA is significantly and positively linked to L2 demotivation (β = 0.314, *p* < .001). Moreover, the findings indicated that R^2^ for L2WTC in online was 0.74 and in the in-person classrooms was 0.82, supporting that 74% of the variance of in-person L2WTC and 82% of the variance of online L2WTC could be explained by this model.

**Table 3:** The direct effect of FLB, FLA, and L2 demotivation on online and in-person L2WTC

	Estimate	p-value	95%CI- lower	95%CI- Upper
FLA→ FLB	− 0.51	< 0.001	− 0.768	− 0.318
FLA→ DM	− 0.21	< 0.001	− 0.455	− 0.102
FLA→ In-person WTC	− 0.25	< 0.001	− 0.432	− 0.117
FLA→ Online WTC	− 0.32	< 0.001	0.510	0.1373
FLB→ DM	− 0.48	< 0.001	− 0.643	− 0.277
FLB→ In-person WTC	− 0.07	< 0.001	− 0.118	− 0.045
FLB→ Online WTC	− 0.04	< 0.001	0.097	0.010
DM→ In-person WTC	− 0.39	< 0.001	− 0.545	− 0.210
DM→ Online WTC	− 0.26	< 0.001	0.432	0.167

*Note* FLA: Foreign Language Anxiety; FLB: Foreign Language Boredom; DM: L2 Demotivation; WTC: Willingness to Communicate

### The Indirect Impacts of FLB, FLA, and L2 Demotivation on in-person L2WTC

FLA had two indirect paths to in-person L2WTC via FLB and L2 demotivation as mediators (see Table 4). First, FLA showed a small yet negative impact on in-person L2WTC through the partial mediation of L2 demotivation (β = −0.137, *p* < .001). Second, FLA had also a small but significant indirect negative impact on in-person L2WTC via the joined effect of the FLB and FLA (β = −0.86, *p* < .01). L2 demotivation also had two indirect paths to in-person L2WTC via FLB and FLA as mediators (see Table 4). First, L2 demotivation had a medium negative impact on in-person L2WTC via the partial mediation of FLB (β = −0.317, *p* < .001). Second, L2 demotivation also had a small yet significant indirect negative link to in-person L2WTC via the joined effect of the FLB and FLA (β = −0.108, *p* < .001). Finally, FLB had two significant indirect paths to in-person L2WTC via the full mediation of L2 demotivation with moderate effect size (β = −0.412, *p* < .001) and a full mediation of the joined small effect of the L2 demotivation and FLA (β = −0.102, *p* < .001).

**Table 4:** Indirect effects of FLA, FLB, and L2 demotivation on online L2WTC

	Estimate	p-value	95%CI- lower	95%CI- Upper
FLA→ FLB→ In-person WTC	− 0.115	0.084	− 0.137	− 0.094
FLA→ DM→ In-person WTC	− 0.137	< 0.001	− 0.163	− 0.112
FLA→ DM*FLB→ In-person WTC	− 0.086	< 0.01	− 0.120	− 0.064
FLB→ FLA→ In-person WTC	− 0.024	0.137	− 0.047	− 0.016
FLB→ DM→ In-person WTC	− 0.412	< 0.001	− 0.513	− 0.326
FLB→FLA*DM→ In-person WTC	− 0.102	< 0.001	− 0.127	− 0.088
DM→ FLA→ In-person WTC	− 0.016	0.251	− 0.021	− 0.009
DM→ FLB→ In-person WTC	− 0.317	< 0.001	− 0.425	− 0.238
DM→ FLA*FLB→ In-person WTC	− 0.108	< 0.001	− 0.214	− 0.087

*Note* FLA: Foreign Language Anxiety; FLB: Foreign Language Boredom; DM: L2 Demotivation; WTC: Willingness to Communicate

### The Indirect Effects of FLB, FLA, and L2 Demotivation on Online L2WTC

FLA had three indirect significant paths to online L2WTC through FLB and L2 demotivation (see Table 5). More specifically, FLA indicated a moderate negative impact on online L2WTC through the partial mediation of FLB (β = −0.426, *p* < .001) and L2 demotivation (β = −0.375, *p* < .001). FLA had also a small but significant indirect negative impact on online L2WTC via the joined effect of the FLB and L2 demotivation (β = −0.141, *p* < .001). L2 demotivation had a small yet significant negative impact on online L2WTC through the partial mediation of FLA (β = −0.176, *p* < .001). L2 demotivation also had a small yet significant indirect negative impact on online L2WTC via the joined effect of the FLB and FLA (β = −0.117, *p* < .001). Although the result did not show any significant direct impact of FLB on online L2WTC, it had a small yet significant indirect path to online L2WTC through the full mediation of FLA (β = −0.148, *p* < .001).

**Table 5:** Indirect effects of FLA, FLB, and L2 demotivation on online L2WTC

	Estimate	p-value	95%CI- lower	95%CI- Upper
FLA→ FLB→ Online WTC	− 0.426	< 0.001	− 0.584	− 0.265
FLA→ DM→ Online WTC	− 0.375	< 0.001	− 0.512	− 0.204
FLA→ FLB*DM→ Online WTC	− 0.141	< 0.001	− 0.198	− 0.089
FLB→ FLA→ Online WTC	− 0.148	< 0.001	− 0.203	− 0.095
FLB→ DM→ Online WTC	− 0.019	0.351	− 0.021	− 0.011
FLB→ FLA*DM→ Online WTC	− 0.018	< 0.001	− 0.034	− 0.012
DM→ FLA→ Online WTC	− 0.176	< 0.001	− 0.219	− 0.137
DM→ FLB→ Online WTC	− 0.022	0.406	− 0.031	− 0.015
DM→ FLA*FLB→ Online WTC	− 0.117	< 0.001	− 0.175	− 0.096

*Note* FLA: Foreign Language Anxiety; FLB: Foreign Language Boredom; DM: L2 Demotivation; WTC: Willingness to Communicate

### Test of Measurement Invariance across Genders

The measurement invariance evaluated for the measurement model across gender mediators (see Table 6). The results demonstrated metric, scalar, and configural invariance across gender (Δχ^2^*p* > .05, ΔCFI ≤ 0.010, ΔRMSEA ≤ 0.015, and ΔSRMR ≤ 0.03).

**Table 6:** Test of measurement invariance for gender

	χ^2^	df	CFI	SRMR	RMSEA	Δχ^2^	p	ΔCFI	ΔSRMR	ΔRMSEA
Gender										
M1: Configural	3306.75	1161	0.927	0.064	0.052	-	-	-	-	-
M2: Metric	3331.12	1142	0.931	0.069	0.054	24.37	0.84	0.004	0.005	0.002
M3: Scalar	3357.63	1087	0.934	0.071	0.055	26.51	0.67	0.003	0.003	0.001

**Note** ∆: differences; χ^2^: chi-square; df: degrees of freedom; TLI: Tucker–Lewis index; CFI: comparative fit index; RMSEA: root mean square error; SRMR: standardized root mean square residual

## Discussion

This study set out to scrutinize the impact of negative affective orientations on L2WTC in the contexts of in-person and online education. Results firstly indicated that FLA and L2 demotivation directly influence EFL learners’ face-to-face and online L2WTC. However, there was no direct path from FLB to in-person and online L2WTC. The findings also revealed that L2 demotivation was the strongest significant predictor of EFL learners’ in-person L2WTC, while FLA was the strongest predictor of learners’ online L2WTC. The results of the study shed light on the elaborate associations between various affective factors and EFL learners’ L2WTC in both in-person and online L2 learning settings. The direct impact of FLA and L2 demotivation on EFL learners’ in-person and online L2WTC highlights the substantial role that negative affective orientations can play in shaping L2 learners’ communication readiness. The finding that L2 demotivation emerges as the strongest negative predictor of in-person L2WTC highlights the harmful consequences of demotivation on L2 learners’ active participation mostly in face-to-face interactions. This aligns with previous research conducted by Khajavy et al. (2018) and Mulyono and Saskia (2021), which indicated the adverse influence of FLA on L2 learners’ willingness to engage in language communication in both in-person and online learning settings. Consistent with the results of the present research, in Peng’s (2015) study, FLA was also identified as one of the strongest direct predictors of L2WTC inside the class. The close link between L2 demotivation and FLA is also mirrored in Choi’s (2017) study, which indicated that these two negative affective orientations can impact L2 learners’ oral communication endeavors.

The findings can be discussed with the focus on the close relationship between L2 engagement and L2WTC (see Gu & Sun, 2021). Given L2 demotivation has been found to influence L2 learners’ engagement (Zhang et al., 2020), the way this negative disposition intertwines with L2WTC sheds light on the complex interplay between affective factors and L2 learners’ overall engagement in communicative interactions in the class. The results can also be interpreted in the light of Self-determination theory (SDT) and the individuals’ psychological need for relatedness, competence, and autonomy. More specifically, these three needs are goal-directed necessities for individuals’ overall psychological well-being, integrity, and personal growth (Deci & Ryan, 2000, 2012). The impact of FLA on online L2WTC aligns with SDT. In online education, EFL learners might exhibit a higher level of anxiety as they might experience a lower sense of relatedness and autonomy in the absence of physical interactions with L2 peers and teachers. Given the academic and personal support provided by L2 teachers and peers have been acknowledged to have a major impact on L2WTC (Khajavy et al., 2018; Wei & Xu, 2022; Zhang et al., 2018), the lack of perceived supports in an online L2 learning environment could potentially increase the anxiety levels among EFL learners and consequently impact their L2WTC negatively. Additionally, congruent with Pekrun’s (2006) control-value theory of achievement emotions, in online setting where EFL learners might have a lower of sense of control over their learning environment and thereby less perceived value for communication, there is a possibility that their L2WTC could be further hindered. This highlights the importance of considering both emotional and motivational factors in designing online L2 learning programs so as to more effectively enhance L2 learners’ online WTC.

Furthermore, although the close impact of L2 motivation on L2WTC in online classes have been acknowledged (see Mulyono & Saskia, 2021), the findings of the present study, revealing a strong and direct impact of L2 demotivation on L2WTC in the context of virtual learning environment, represent a novel contribution to the field of L2 education. The finding is particularly significant due to the limited exploration of negative affective factors like L2 demotivation in shaping online L2WTC of EFL learners (Eddy-U, 2015). Additionally, in the current study, insufficient class materials and demotivating class environment were the strongest antecedents of L2 demotivation. This corresponds with Dörnyei’s (1998) study, where insufficient school facilities and uninteresting learning materials were among the precursors of L2 demotivation. Zhang et al.’s (2020) study also indicated that demotivating classroom materials can influence FLA and indirectly EFL university learners’ intention to continue the courses. These findings implicate that when L2 learners perceive classroom materials as demotivating, it can trigger an increase in their FLA, which in turn indirectly affects the intention of EFL university learners to continue their courses. It is also noteworthy that previous research has shown the strong relationship between L2 demotivation and FLA (Choi, 2017; Gao & Liu, 2022). Thus, the findings in the present study contribute to the broader understanding of how these affective factors jointly interact and influence EFL learners’ communicative willingness in the class. Furthermore, given most of the demotivating factors identified in prior research are linked to different elements of the classroom environment that are influenced by the teacher (Dörnyei, 2022), the prominence of L2 teachers in fostering communication willingness of EFL learners cannot be underestimated. Thus, the pivotal role of L2 teachers in alleviating the demotivating factors and cultivating a positive classroom environment that fosters communication willingness appears to be crucial in the dynamics of L2 classes.

In contrast to Zhang et al.’s (2022) study, which suggests a significant and direct influence of FLB on EFL students’ WTC in the class, in the current study, FLB did not have a direct effect on communication willingness of EFL learners. However, FLB exhibited indirect mediation paths to L2WTC through FLA and L2 demotivation. The underlying mechanism for the indirect impact of FLB on L2WTC can be explained through the lens of El Deen’s (2023) study, where L2 demotivation was an antecedent of FLB, indicating that L2 demotivation yields FLB which subsequently influences L2WTC indirectly. Given there is a scarcity of research on the interplay between FLB and L2 demotivation in L2 research (Pawlak et al.,2020a), the findings might provide valuable insights into understanding the complex relationship between these two constructs and their joined effect on L2WTC. Moreover, the absence of a direct link between FLB and L2WTC in either setting suggests that while L2 boredom might contribute to the learners’ overall engagement and motivation (Derakhshan, Fathi, et al., 2022; Lan et al., 2023), it might not directly inhibit their communication willingness. The indirect impact of FLB on L2WTC has also been indicated by Lan et al.’s (2023) study, where FLB moderated the direct link between L2 motivational intensity and L2 WTC as well as the slope between L2 enjoyment and L2 WTC. The indirect impact of FLB on L2WTC via the joined effect of other affective factors highlights the intricate amalgamation of emotional dimensions in shaping EFL learners’ online and in-person WTC. All these studies underscore the concealed, yet pervasive, nature of L2 boredom in overall L2 learning and specifically in L2WTC.

### Implications

Several pedagogical considerations can be offered based on the findings: Firstly, emotion-focused interventions should be integrated into L2 learning environments to alleviate negative emotional experiences such as FLA and L2 demotivation and thereby improve EFL learners’ communication willingness in both face-to-face and online settings. Given the academic and personal supports provided by L2 teachers and peers have been confirmed to foster L2WTC and speaking motivation in both online and in-person classes (see Solhi, 2023; Wei & Xu, 2022), educational institutions should invest in fostering collaborative and supportive learning communities that encourage positive interactions between L2 learners, peers, and teachers (Derakhshan et al., 2023). Moreover, recognizing the pivotal role of autonomy and relatedness in emotional well-being (Ryan & Deci, 2000, 2012), curricula and instructional approaches could be designed to empower L2 learners with a sense of control over their learning experiences while fostering a sense of belongingness within the L2 community. For example, by integrating opportunities for L2 learners to engage in self-directed activities, set goals, and make choices in their learning paths, L2 instructors can ingrain a sense of control and autonomy. Additionally, instructional approaches that emphasize collaborative peer/group activities and interactions, and joint problem-solving tasks can cultivate a strong sense of relatedness within the L2 learning community. Given sense of belonging and relatedness in L2 educational settings is closely associated with L2 speaking motivation (Solhi, 2023), these approaches have the potential to not only enhance learners’ emotional well-being and intrinsic motivation but also to foster communication willingness in face-to-face and online learning environments.

To cultivate a sense of connectedness in online L2 classes and consequently to foster online L2WTC, L2 instructors can establish supportive virtual learning environments that stimulate L2 speaking motivation. This can be achieved through utilizing diverse teaching modalities such as visual aids, delivering constructive feedback during sessions, designing collaborative peer and group tasks, encouraging reflective learning practices to obtain input from learners, and selecting captivating subjects. Additionally, the implementation of collaborative online projects emerges as a viable strategy. Peer tutoring, which involves individuals from similar social backgrounds aiding each other’s learning while refining their own understanding through teaching (Topping, 1996), can be a particularly effective approach. More specifically, integrating peer tutoring initiatives in online classes encourages L2 learners to form collaborative groups in virtual rooms, where they can collaboratively assist each other. Such peer tutoring can potentially strengthen their comprehension through the act of teaching in a mutually supportive milieu. In this way, the supports provided by L2 peers can foster their sense of relatedness in online environments and reduce FLA that hinders online L2WTC.

### Limitations and Suggestions for Future Studies

The findings of the present study need to be considered with the limitations. Firstly, the focus of the study was on the impact of only the negative affective orientations on face-to-face and online L2WTC. Future research could consider testing the model with the integration of positive affective orientations such as L2 enjoyment. The impact of different personality traits such as L2 girt on L2WTC with the mediating roles of FLB, FLA, L2 demotivation can be intriguing research avenue. In the present study, while the construct of L2 demotivation was multifaceted, FLB and FLA were examined as distinct single factors contributing to EFL learners’ L2WTC. Considering the complex nature of affective L2 orientations, future studies could investigate a more comprehensive framework that explores the interplay between various dimensions of FLB and FLA along with other affective factors. In addition, the focus of the current study was on EFL learners at higher education, testing the model with k-12 students would provide valuable insights into the generalizability and applicability of the findings across various educational levels. Besides, examining the role of demographic variables such as gender and school type could provide a deeper understanding of how they interact with affective factors and influence learners’ L2WTC across different settings. Furthermore, exploring the effectiveness of targeted interventions aimed at alleviating negative affective orientations and enhancing L2 communication willingness could offer practical insights for L2 educators and curriculum developers seeking to promote L2WTC in both online and in-person environments.

## Conclusion

In conclusion, the study indicated that EFL learners’ levels of anxiety and demotivation can adversely influence their in-person and online communication willingness, with L2 demotivation being the strongest negative predictor of face-to-face L2WTC. In addition, L2 anxiety was identified as the strongest negative predictor of online L2WTC. While L2 boredom had an indirect path to L2WTC, it exhibited no direct impact on communication willingness, either online or in-person. The findings highlight the complex interplay between affective orientations and L2WTC in various learning settings and provide insights into the ways in which these adverse emotional factors can influence EFL learners’ communication willingness based on the learning context. Lastly, the findings help discern the underlying the dynamics of L2WTC in different L2 learning environments.

## Data Availability

The data and materials can be provided upon a reasonable request.
